# Time-Course Changes of Steroidogenic Gene Expression and Steroidogenesis of Rat Leydig Cells after Acute Immobilization Stress

**DOI:** 10.3390/ijms151121028

**Published:** 2014-11-14

**Authors:** Han Lin, Kai-ming Yuan, Hong-yu Zhou, Tiao Bu, Huina Su, Shiwen Liu, Qiqi Zhu, Yiyan Wang, Yuanyuan Hu, Yuanyuan Shan, Qing-quan Lian, Xiao-yun Wu, Ren-shan Ge

**Affiliations:** 1 Department of Anesthesiology of the Second Affiliated Hospital; Wenzhou Medical University, Wenzhou 325027, Zhejiang, China; E-Mails: linhanwenzhou@wzhealth.com (H.L.); yuankaiming@aliyun.com (K.-m.Y.); butiao999@gmail.com (T.B.); suhuinawz@gmail.com (H.S.); shiwenliu77@gmail.com (S.L.); zhuqiqi998@gmail.com (Q.Z.); wangyiyan1988@gmail.com (Y.W.); huyuanyuan889@gmail.com (Y.H.); 2 Department of Pharmacology of School of Pharmacy, Wenzhou Medical University, Wenzhou 325035, Zhejiang, China; E-Mail: zhouhy@wmu.edu.cn; 3 Research Academy of Reproductive Biomedicine, Wenzhou Medical University, Wenzhou 325000, Zhejiang, China; E-Mail: shanyuanyuan6@gmail.com; 4 Huzhou Maternity & Child Care Hospital, Huzhou 313000, Zhejiang, China

**Keywords:** acute stress, Leydig cell, steroidogenic enzymes, rat, corticosterone, StAR

## Abstract

Leydig cells secrete testosterone, which is essential for male fertility and reproductive health. Stress increases the secretion of glucocorticoid (corticosterone, CORT; in rats), which decreases circulating testosterone levels in part through a direct action by binding to the glucocorticoid receptors (NR3C1) in Leydig cells. The intratesticular CORT level is dependent on oxidative inactivation of glucocorticoid by 11β-hydroxysteroid dehydrogenase 1 (HSD11B1) in Leydig cells. In the present study, we investigated the time-course changes of steroidogenic gene expression levels after acute immobilization stress in rats. The plasma CORT levels were significantly increased 0.5, 1, 3 and 6 h after immobilization stress, while plasma testosterone levels were significantly reduced 3 and 6 h, after stress and luteinizing hormone (LH) did not change. Immobilization stress caused the down-regulation of *Scarb1*, *Star* and *Cyp17a1* expression levels in the rat testis starting at the first hour of stress, ahead of the significant decreases of plasma testosterone levels. Other mRNA levels, including *Cyp11a1*, *Hsd3b1* and *Hsd17b3*, began to decline after 3 h. *Hsd11b1* and *Nos2* mRNA levels did not change during the course of stress. Administration of glucocorticoid antagonist RU486 significantly restored plasma testosterone levels. In conclusion, *Scarb1*, *Star* and *Cyp17a1* expression levels are more sensitive to acute stress, and acute immobilization stress causes the decline of the steroidogenic pathway via elevating the levels of glucocorticoid, which binds to NR3C1 in Leydig cells to inhibit steroidogenic gene expression.

## 1. Introduction

Leydig cells secrete the male steroid hormone, testosterone, which stimulates differentiation of the male phenotype and spermatogenesis in the testes. In the male rat, Leydig cells are one of two cell types specialized for the synthesis of steroids, the other being the adrenocortical cells of the adrenal gland. The steroid biosynthetic pathways in both glands use a common precursor, cholesterol. Cholesterol in lipoprotein-bound form is transported via scavenger receptor class B member 1 (SCARB1, encoded by *Scarb1*) into both cells, where cholesterol is released. In the Leydig cell, after stimulation by luteinizing hormone (LH), cholesterol is mobilized to the inner mitochondrial membrane by the steroidogenic acute regulatory protein (StAR, encoded by *Star*) [1], where it is converted to the 21-carbon steroid, pregnenolone, by cytochrome P450 side chain cleavage enzyme (CYP11A1, encoded by *Cyp11a1*). Pregnenolone diffuses out of the mitochondrion into the membranes of the smooth endoplasmic reticulum, which contains 3β-hydroxysteroid dehydrogenase 1 (HSD3B1, encoded by *Hsd3b1*). Upon conversion of pregnenolone into progesterone by dehydrogenation at the three-carbon and Δ^5→4^ isomerization, two additional smooth endoplasmic reticulum enzymes, cytochrome P450 17α-hydroxylase/C_17–20_ lyase (CYP17A1, encoded by *Cyp17a1*) and 17β-hydroxysteroid dehydrogenase isoform 3 (HSD17B3, encoded by *Hsd17b3*), convert progesterone sequentially to 17-hydroxyprogesterone, androstenedione and, ultimately, to testosterone, a 19-carbon steroid [2]. In the cortical cells of the rat adrenal gland, glucocorticoid corticosterone (CORT) is synthesized.

The testosterone production by Leydig cells is predominantly regulated by pituitary LH, which contains two subunits: The LH α chain and the LH β chain (LHB, encoded by *Lhb*), which are rate-limiting. LH binds to the LH receptor (LHCGR, encoded by *Lhcgr*) on the surface of the Leydig cell to activate adenylyl cyclase, leading to a cascade to stimulate testosterone production. In the pituitary, LH secretion is positively regulated by gonadotropin-releasing hormone (GnRH), which binds to its receptor (GnRHR, encoded by *Gnrhr*) and is negatively regulated by estradiol, which binds to its receptor α (ESR1, encoded by *Esr1*), or testosterone [3].

These two steroidogenic cell types (cortical cells and Leydig cells) interact, because of the presence of the glucocorticoid receptor (NR3C1, encoded by *Nr3c1*) in the Leydig cells [4,5]. In this case, the glucocorticoid hormones secreted by the adrenal gland can act on Leydig cells to elicit their effects. In the case of stress, the adrenal glands secrete glucocorticoid hormones and epinephrine into the bloodstream to initiate responses to counter stress. An over-abundance of glucocorticoid and epinephrine activities is a hallmark of stress [6,7,8]. The ability of stress to suppress the reproductive system is well recorded [6,9]. A significant reduction of plasma testosterone level is one of the signs of acute immobilization stress. An acute stress-mediated decrease of testosterone production has been found to be related to the suppression of the activities of testicular steroidogenic enzymes. It is well established that acute stress-increased glucocorticoid activity appears involved in the reduced testosterone levels in both acute and chronic stress stimulation [9] and that the increased activity of epinephrine seems more involved in the suppression of testosterone production after prolonged stress stimulation [10,11]. A direct NR3C1-mediated inhibition of testosterone biosynthesis by glucocorticoid has been proposed [12]. Stress increases plasma glucocorticoid, and a number of* in vitro* studies have shown that glucocorticoid directly inhibits testosterone production by Leydig cells [12,13]. Although it is well established that acute stress increased glucocorticoid activity and reduced testosterone levels, the time-course changes of Leydig cell steroidogenic messenger RNAs, and which steroidogenesis-related protein is more sensitive to the stress-mediated inhibition of, have not been sufficiently examined. In addition, whether 11β-hydroxysteroid dehydrogenase isoform (HSD11B1, encoded by *Hsd11b1*), which inactivates glucocorticoids and controls the level of active glucocorticoid in Leydig cells [4,14,15,16], is affected during the course of stress is also unknown. In the present study, we investigated time-course changes of the androgen-biosynthetic system and the expression levels of several glucocorticoid targeted genes.

## 2. Results

### 2.1. Plasma Hormone Levels

**Figure 1:**
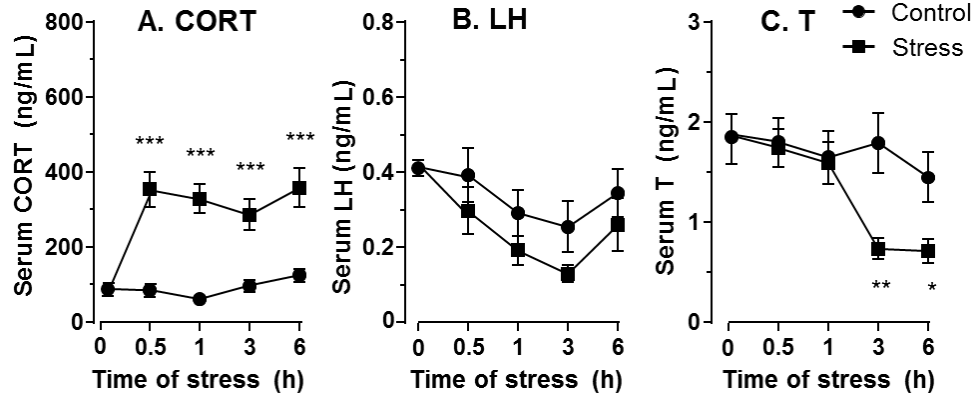
Plasma corticosterone (CORT), luteinizing hormone (LH) and testosterone (T) levels during the course of stress. (**A**) Plasma CORT; (**B**) Plasma LH; and (**C**) Plasma T levels. Mean ± SEM (*n* =12–25, each time point). *n* = 12 for 0 h group, in which animals of the control and stress group were calculated together. *, ** and *** indicate significant difference compared to control (unstressed) at each time point at *p* < 0.05, *p* < 0.01 and *p* < 0.001, respectively.

**Figure 2:**
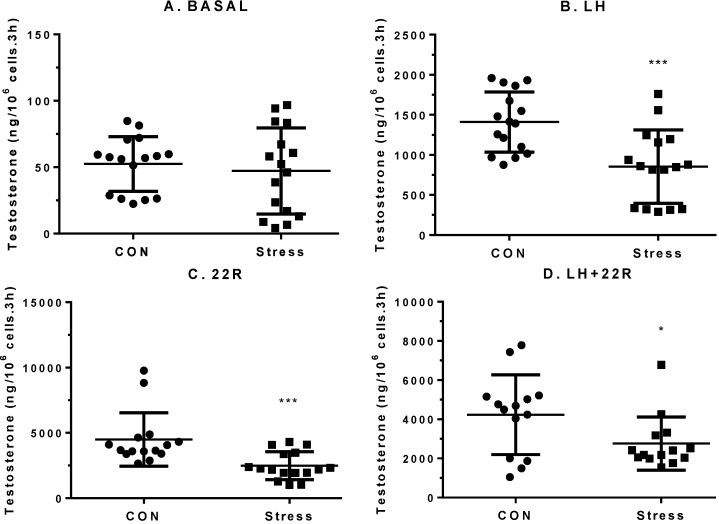
Testosterone production by rat Leydig cells from control (CON, unstressed) and stress rats. Leydig cells were stimulated for 3 h without (basal) or with 100 ng/mL LH (LH) or 20 µM 22*R*-hydroxycholesterol (22*R*) or both (LH + 22*R*). (**A**) Basal; (**B**) LH; (**C**) 22*R*; and (**D**) LH + 22*R*. Mean ± SEM (*n* = 14–16). *, *** indicate significant differences compared to the control at *p* < 0.05 and *p* < 0.001, respectively.

The average values of plasma CORT in the control rats had no significant changes between groups (Figure 1A). During the course of immobilization stress, plasma CORT levels in the stressed rats were significantly increased 0.5, 1, 3 and 6 h (*p* < 0.001) after being restrained in the mesh compared to the control rats (the CORT value at 0 h = 92.02 ± 23.83 ng/mL, mean ± SEM (standard error of the mean), Figure 1A), indicating that these rats were under stress 0.5, 1, 3 and 6 h after being in the mesh. Plasma LH levels in these rats did not change significantly during the course of stress in all groups (Figure 1B). At 0 h, the serum testosterone level was 1.92 ± 0.19 ng/mL (mean ± SEM, *n* = 12). Since there is a circadian nature to plasma testosterone, the plasma testosterone levels under stress were compared to the respective control time point. However, plasma testosterone levels were significantly lower compared to control rats 3 (*p* < 0.01) and 6 h (*p* < 0.05) after being in the mesh (Figure 1C), suggesting that the primary cause of lower testosterone is from testis and not from pituitary LH secretion. We further isolated Leydig cells from the control rats and the stressed rats under 6 h immobilization stress and incubated these cells in the absence or the presence of 100 ng/mL LH, or 20 µM 22*R*-hydroxycholesterol, or both. Immobilization stress did not inhibit basal testosterone production, but significantly inhibited LH (*p* < 0.001), 22*R*-hydroxycholesterol (*p* < 0.001) and LH plus 22*R*-hydroxycholesterol (*p* < 0.05) mediated testosterone production of Leydig cells (Figure 2), confirming that the reduced plasma levels of testosterone were caused by the defects of Leydig cell steroidogenesis.

### 2.2. Gene Expression Levels of Pituitary and Testes

**Figure 3:**
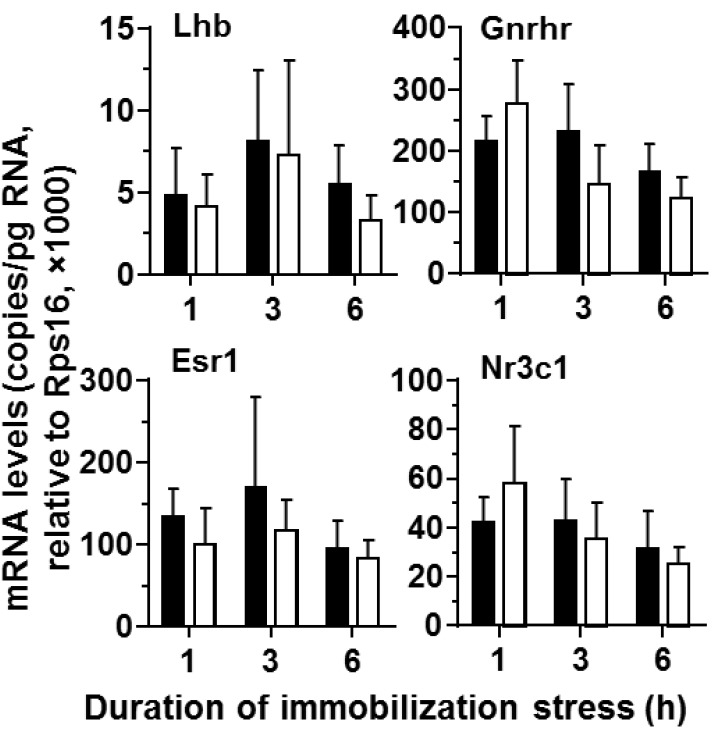
Messenger RNA levels of pituitaries from control and stressed rats during the course of immobilization stress. Black bars indicate control and white bars indicate immobilization stress. Mean ± SEM (*n* = 6). No significant difference was found between the control and the stressed groups.

The most examined genes are Leydig cell-specific genes, including *Lhcgr*; therefore, the gene expression using whole testes can represent Leydig cell function. As shown in Figure 3, the pituitary *Lhb* level was not affected during the course of stress, confirming the changes of plasma LH levels. Furthermore, the regulatory receptor genes, including *Gnrhr*, *Esr1* and *Nr3c1*, were not affected by the stress.

**Figure 4:**
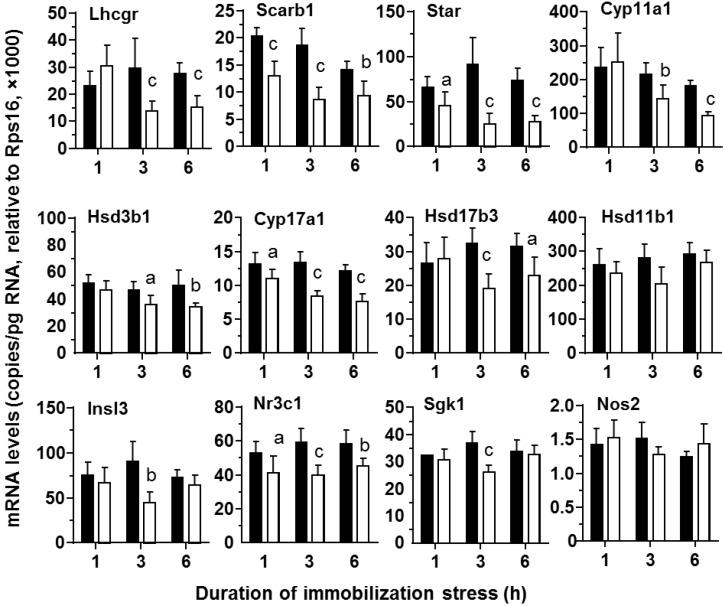
Messenger RNA levels of testes from control and stressed rats during the course of immobilization stress. Control (black bar) and immobilization stress (white bar). Mean ± SEM (*n* = 6). Letters a, b, and c indicate significant difference compared to the control at each time point at *p* < 0.05, *p* < 0.01 and *p* < 0.001, respectively.

In contrast, in the testis, *Scarb1* (*p* < 0.001), *Star* (*p* < 0.01) and *Cyp17a1* (*p* < 0.05), were significantly down-regulated 1 h after immobilization stress (Figure 4). The other steroidogenic genes, including steroidogenic enzymes *Cyp11a1* (*p* < 0.01), *Hsd3b1*, (*p* < 0.05) and *Hsd17b3* (*p* < 0.001), as well as LH receptor *Lhcgr* (*p* < 0.001)*,* were significantly down-regulated 3 h after these rats were restrained in the mesh (Figure 4). The expression levels of most genes examined continued to be lower 6 h after stress (*Lhcgr*, *Scarb1*, *Star*, *Cyp11a1*, *Hsd3b1*, *Cyp17a1*, *Hsd17b3*).

### 2.3. Levels of StAR Protein and Steroidogenic Enzymes

**Figure 5:**
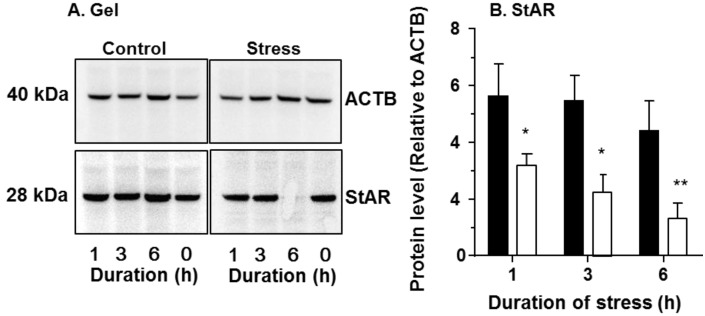
Protein levels of steroidogenic acute regulatory protein (StAR) of testes from control and stressed rats during the course of stress (1 to 6 h). (**A**) The representative gel; and (**B**) The semi-quantitative levels of StAR, with control (black bar) and immobilization stress (white bar). Protein levels were adjusted by β-actin (ACTB). Mean ± SEM (*n* = 6). *, ** indicate significant difference compared to the control at each time point at *p* < 0.05 and *p* < 0.01, respectively.

**Figure 6:**
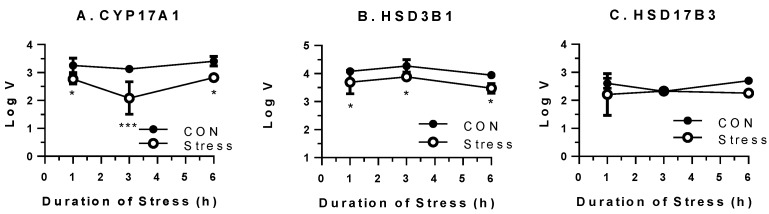
Steroidogenic enzyme activities in the testes from control and stressed rats during the course of immobilization stress. (**A**) 17α-hydroxylase/17,20-lase (CYP17A1) to catalyze progesterone (P4) to androstenedione (D4); (**B**) 3β-hydroxysteroid dehydrogenase 1 (HSD3B1) to catalyze pregnenolone to P4; and (**C**) 17β-hydroxysteroid dehydrogenase 3 (HSD17B3) to catalyze D4 to testosterone (T). Mean ± SEM (*n* = 6). LogV = log velocity (µg/mg protein.min). *, *** indicate significant difference compared to control (CON) at each time point at *p* < 0.05 or *p* < 0.001, respectively.

We further performed western blot for StAR protein (Figure 5) and steroidogenic enzyme activities for CYP17A1, HSD3B1 and HSD17B3 (Figure 6). As shown in Figure 5 and Figure 6, the StAR protein level and CYP17A1 activity were significantly down-regulated 1–6 h after being put in the stress mesh. The HSD3B1 activities were lower starting 3 h under stress (Figure 6).

### 2.4. Antagonism by Glucocorticoid Receptor Antagonist RU486

**Figure 7:**
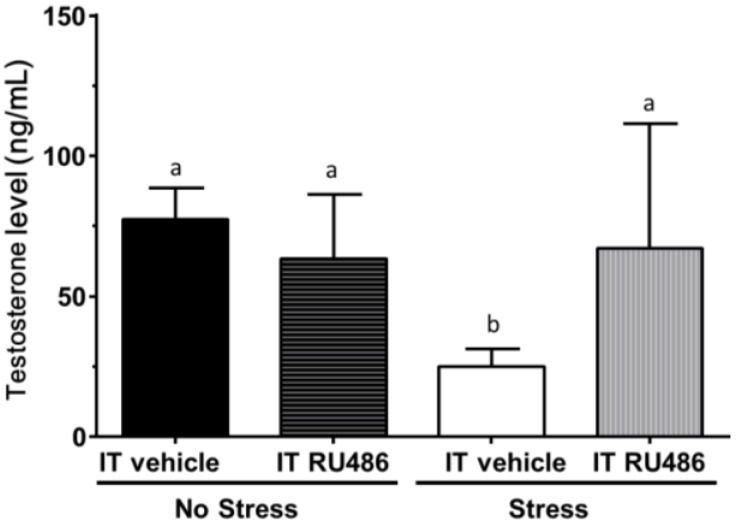
Interstitial fluid testosterone levels. Rats were assigned to no stress/intratesticular (IT) injection of vehicle (no stress/IT vehicle), no stress/intratesticular injection of RU486 (no stress/IT RU486), immobilization stress/Intratesticular injection of vehicle (stress/IT vehicle) and immobilization stress injected with RU486 (stress/IT RU486) groups. Mean ± SEM (*n* = 6). Identical letters indicate no significant difference between two groups at *p* < 0.05.

We asked whether the increased CORT levels suppressed testosterone production via NR3C1. We injected rats with RU486, an antagonist of NR3C1, and then, the rats were subjected to immobilization stress. As shown in Figure 7, RU486 significantly reversed the stress-mediated suppression of testosterone production.

## 3. Discussion

Stress is a widespread condition, and stressors are increasingly present in modern society, among them noise, over-crowding and pollution, leading to health problems that all trace back to stress as a contributing cause. One stress-related health problem is low testosterone, which may further contribute to the reduction of spermatogenesis. In humans, the severe psychological stress brought on by the death of a relative or spouse consistently lowered sperm count [17]. Stress was recognized by clinicians as a contributing factor to human infertility, and couples needing assisted reproduction are now counseled in measures to reduce stress [18,19,20,21].

The mechanism of suppressing testosterone biosynthesis by stress may depend on the stress duration. The mechanisms of acute and chronic stresses in testosterone biosynthesis may be different. Indeed, a repeated immobilization stress (10 days) has been shown to inhibit steroidogenesis of Leydig cells, induce Leydig cell apoptosis [22,23] and increase adrenergic receptors [11], and this adverse effect after chronic stress can be partially antagonized by the blockade of α₁-adrenergic receptors [10].

In this study, immobilization stress to male rats resulted in an increased plasma CORT level as earlier as 30 min under the immobilization stress. Reduced plasma testosterone levels followed 3 h after being subjected to the stress. The reduction of testosterone production followed significant initial down-regulation of cholesterol transporter (*Scarb1* and *Star*) levels and steroidogenic enzyme (*Cyp17a1*) starting 1 h after acute stress.

The significant increase of CORT levels in the immobilization stressed rats 0.5 to 6 h after being subject to the immobilization stress is an indicator of the stress response. This corroborates the significant increase in total serum CORT levels in the stressed rats under other types of stresses [24,25]. The amount of the bioavailable glucocorticoid depends on the circulating CORT level, the plasma CORT binding globulin concentration and local CORT metabolism by 11β-hydroxysteroid dehydrogenase 1 (HSD11B1) in rat Leydig cells. In rat Leydig cells, HSD11B1 is the predominant isoform [16], and it acts primarily as an oxidase [15,26] to protect Leydig cells from the adverse effects of CORT [27]. The other isoform is HSD11B2, which also acts an oxidase, and only accounts for the 1/1000th of the total amount of Leydig cell 11β-hydroxysteroid dehydrogenase [16]. However, in the present study, we did not observe any change of *Hsd11b1* expression level during the course of stress. Thus, the excess of CORT without the increase of *Hsd11b1* expression could lead to the increased intracellular level of CORT, which inhibits Leydig cell steroidogenesis. It has been demonstrated that glucocorticoid inhibits Leydig cell steroidogenesis by targeting many steroidogenic proteins, including *Cyp11a1* [12], *Star* [28,29] and *Hsd3b1* [29]. Since StAR is the rate-limiting protein for steroid production in the steroidogenic cells [1,30], the inhibition of its expression causes the suppression of androgen biosynthesis. Martin and Tremblay revealed that the molecular mechanism of glucocorticoid on the suppression of *Star* expression is at least involved in the suppression of NR4A1-dependent transactivation of the *Star* promoter in mouse MA-10 Leydig cells by reducing NR4A1 (not NR5A1) recruitment to the proximal *Star* [28].

During the course of stress, the serum testosterone levels of the stressed rats were lowered significantly 3–6 h after being subject to the immobilized stress (Figure 1C). The reduction of serum testosterone levels may follow the reduction of the secretion of testosterone from Leydig cells, especially as serum LH levels were not suppressed. Thus, CORT may directly inhibit Leydig cell steroidogenesis. We observed the significant down-regulation of cholesterol transportation (*Scarb1* and *Star*) and steroidogenic enzyme (*Cyp17a1*) as early as the first hour of stress (Figure 4), followed by steroidogenic enzymes (*Cyp11a1*, *Hsd3b1* and *Hsd17b3*) later on (Figure 4). The dramatic down-regulation of many steroidogenesis-related genes, including *Star*, 3 h under stress could explain the significant reduction of testosterone secretion. After 3–6 h under stress, the *Lhcgr* expression level was also significantly down-regulated. This down-regulation of *Lhcgr* might explain the significant reduction of testosterone secretion from Leydig cells after LH stimulation (Figure 2). Interestingly, the expression level of one gene (*Scarb1*) changed across time, while others did not. Because there is circadian change for testosterone biosynthesis, there is a possibility that change of *Scarb1* follows circadian patterns.

In Figure 1, CORT was up at 30 min and StAR was down at 1 h. However, testosterone was decreased until 3 h. It appears that there are two phases after acute stress. The first phase represents a rapid decrease in StAR, which would result in a parallel decrease in testosterone, followed by a somewhat slower decrease in other steroidogenic enzymes, such as CYP11A1, HSD3B1 and CYP17A1, which would also inhibit the production of testosterone. The significant decrease of testosterone level at 3 h of acute stress could be contributed to by the combination of down-regulation of both StAR and these steroidogenic enzymes.

Several stress-induced factors mediating the effects on Leydig cells have been postulated to reduce testosterone production. These include CORT [31,32,33,34], testicular opioids [35] or nitric oxide (NO) [36]. NO is a reactive free radical that acts as a biologic mediator in several processes. It is synthesized by nitric oxide synthase (NOS). There are several isoforms of NOS, and inducible NOS (encoded by *Nos2*) is present in rat Leydig cells [37]. NOS2 synthesizes NO, which is a reactive free radical, acting as a biologic mediator in several processes [38]. Several studies debate the possible involvement of NOS signaling in acute stress-mediated suppression of Leydig cells [28,38,39]. However, knockout of several NOS isoforms did not antagonize the stress-mediated action on testosterone production [38,39], suggesting that NO signals in the acute stress-mediated action are minimal. Indeed, in the present study, we also did not observe the significant increase of the expression level of the inducible NOS (*Nos2*) (Figure 4). The acute stress-induced suppression of testosterone production in the Leydig cells could be mediated by the glucocorticoid receptor (NR3C1). Indeed, the intratesticular injection of NR3C1 antagonist, RU486, completely prevented the interstitial fluid testosterone level change against the immobilization stress (Figure 7), suggesting that CORT acts via the NR3C1 in the Leydig cells. Serum and glucocorticoid-regulated kinase 1 (SGK1, encoded by *Sgk1*) is a serine/threonine kinase that is acutely regulated by glucocorticoids [40]. Therefore, stress may affect the expression levels of *Sgk1*. We measured the expression levels of *Nr3c1* and its target gene *Sgk1* and found that there were decreased expression levels of *Nr3c1* at ≥1 h and that of *Sgk1* 3 h under the stress (Figure 4), suggesting a compensatory mechanism to minimize CORT action [27].

## 4. Materials and Methods

### 4.1. Chemicals

Rat LH standard NIDDK-r-LH-I9 and LH antibody NIDDK-anti-rLH-S-11 were obtained through the National Hormone and Pituitary Program (Rockville, MD, USA). Radioactive ^125^I-rat LH was obtained through Covance Laboratories (Vienna, VA, USA), and IgG antiserum was obtained from ICN Pharmaceuticals (Costa Mesa, CA, USA). ^3^H-CORT, ^3^H-pregnenolone, ^3^H-progesterone, ^3^H-androstenedione, ^3^H-CORT and ^3^H-testosterone were purchased from DuPont-New England Nuclear (Boston, MA, USA). The CORT antiserum B3-163 was obtained from Endocrine Sciences (Calabasas, CA, USA). NAD^+^ and NADPH were purchased from Sigma (St. Louis, MO, USA).

### 4.2. Animals

Adult male Sprague Dawley rats (250–300 g body weight) were purchased from Shanghai Animal Center (Shanghai, China) and housed under controlled environmental conditions (temperature 22 ± 2 °C; 12:12 h light: dark, with lights on from 600 to 1800 h). All animals had *ad libitum* access to food and water. All animals were handled to adapt for at least three weeks prior to the beginning of the experiment. The animals were housed in IVC cages (one rat per cage) on soft chip bedding and provided pellet chow (Shanghai Laboratory Animal Center). All animal procedures were performed in accordance with the National Institutes of Health Guide for the Care and Use of Laboratory Animals according to protocols approved by the Animal Care and Use Committee of Wenzhou Medical University.

### 4.3. Rat Stress Model: Time Course Experiment

#### 4.3.1. Experiment 1

Rats were transported from their home room into a procedure room, and acute immobilization stress was performed as previously described [39]. The animals were placed in wire mesh restrainers and secured at the head and tail ends with clips, as described [39]. The procedure effectively restricted movement. Control rats were left in the home room and anesthetized prior to transfer to the procedure room for perfusion. The start of immobilization stress began at 9 AM and the treatment durations were 0, 0.5, 1, 3 and 6 h (*n* = 12–25 per time point). Control animals were left undisturbed in their cages for the duration of the experiment and sampled at the same time points. At the end of each stress period, animals were rapidly decapitated by a guillotine either immediately without being stressed (0 h) or 0.5, 1, 3 and 6 h after the restraint stress. Before, the animals were placed in a dry ice container and euthanized by CO_2_ for 1 min. Then, the animals were decapitated by a guillotine, and trunk blood was collected in tubes containing heparin and centrifuged at 500× *g*. The plasma was stored at −20 °C until assay. Testes were removed and stored at −70 °C. Testes were used for the analysis of the Leydig cell-specific gene expression and protein levels. The overall design was replicated.

To investigate whether Leydig cell steroidogenesis was affected after stress, another set of animals (*n* = 6 per group) were subjected to immobilization stress for 6 h, as described above, since at this time point, plasma testosterone levels were significantly reduced after our pilot study. At the end of the stress period, animals were euthanized by CO_2_, and trunk blood was collected as described above for hormonal assays. Leydig cells were harvested for direct measurement of steroidogenesis* ex vivo* as follows.

#### 4.3.2. Experiment 2

To investigate the involvement of NR3C1 in the glucocorticoid-induced testosterone decrease during acute stress, RU486 (17(-hydroxy-11(-(4-dimethy-aminophenyl-1)-17)-(1-prop-1-ynyl)-oestra-4, 9-diene-3-one, (Roussel Uclaf S.A., Paris, France) was administered* in vivo* by intratesticular injection prior to the stress session. The dose (16 µg) of RU486 was selected based on a previous study conducted in rats [31]. RU486 was first dissolved in absolute ethanol and subsequently diluted with the vehicle, 45% aqueous 2-hydroxypropyl-cyclodextrin (Catalog Number 0926, Sigma), to attain the needed concentrations (the final concentration of ethanol was 0.8%, which did not affect Leydig cell function [31]). Animals (*n* = 6 per group) were divided into 4 groups: Non-stress group, non-stress group of rats injected intratesticularly with RU486, stress group and stress group of rats injected with RU486. Rats were subjected to immobilization stress for 6 h, as described above. At the end of the stress period, animals were euthanized by CO_2_, testes were taken and interstitial fluids were prepared according to the previously described method [41]. In brief, several holes were punctured in the rat testis using needles and placed into centrifuge tubules, and the testis was centrifuged at 800× *g* for 10 min to collect interstitial fluids.

### 4.4. Leydig Cell Isolation and Leydig Cell Steroidogenesis Ex Vivo

Leydig cells were purified as described [42]. In brief, testes were removed, decapsulated and dispersed in 10 mL of medium 199 (M-199, Sigma) with 0.25 mg/mL collagenase (Sigma) in a shaking water bath at 34 °C for 10 min. To terminate collagenase dispersion, 1% bovine serum albumin (BSA, Sigma) M-199 buffered with 15 mM 4-(2-hydroxyethyl)-1-piperazineethanesulfonic acid (HEPES, Sigma) and 4 mM sodium bicarbonate, and 25 µg/mL soybean trypsin inhibitor were added to dilute the original suspension 1:5. Tubes were then capped and inverted several times. Seminiferous tubules were allowed to settle, and the supernatant containing the interstitial cells was collected by aspiration. The tubes containing the settled seminiferous tubules were refilled with 1% BSA, and the procedure was repeated several times to further harvest the interstitial cells. The cells were pelleted in 250 mL tubes by centrifugation at 800× *g* for 20 min at 4 °C and then fractionated using a continuous Percoll gradient (55% Percoll in Hanks balanced salt solution). The gradients were formed* in situ* by centrifugation at 220× *g* for 30 min at 10 °C. A tube containing density marker beads and 35 mL of 55% Percoll solution was used as a reference. Leydig cells were recovered starting at a density of 1.07 mg/mL to the top of the red blood cell layer. Leydig cells were washed using M-199 and were pelleted at 200× *g* for 10 min at 4 °C. The Leydig cell purity was approximately 97.1% ± 2.1% (*n* = 6), as determined by histochemical staining for HSD3B1 using 0.4 mM etiocholan-3-ol-17-one as the enzyme substrate as described (see Figure S1) [43]. Isolated adult Leydig cells were studied* ex vivo*. Leydig cells were incubated at a concentration of 0.1 × 10^6^ cells/mL in the culture medium consisting of DMEM and Ham’s F-12 medium (D2906, Sigma) buffered with 15 mM HEPES and 14 mM NaHCO_3_ and containing 1% BSA for 3 h at 34 °C in a shaking water bath. Incubations of quadruple samples were conducted in medium alone (basal) or in medium plus a maximally stimulating dose of ovine LH (100 ng/mL), 20 µM 22-*R*-hydroxysteroid cholesterol (22*R*) or LH (100 ng/mL) plus 20 µM 22*R*. At the end of 3 h, the samples were centrifuged at 500× *g*. Supernatants were used to measure testosterone levels by radioimmunoassay (RIA).

### 4.5. RIA of CORT, LH and Testosterone

The plasma CORT level was measured using the method from Spencer* et al.* [24]. In brief, plasma samples (20 µL) were diluted in 1 mL of 0.01 M phosphate buffer and were heated at 60 °C for 1 h in order to inactivate corticosteroid-binding globulin (CBG). The heat-inactivated samples (100 µL in triplicate) were incubated overnight with a mixture of rabbit CORT antiserum and ^3^H-CORT. CORT standards (10–2000 pg/100 µL) were assayed in parallel. Bound steroid was separated from free steroid by mixing with dextran-coated activated charcoal followed by centrifugation. The bound supernatant was put into a bottle with scintillation cocktail, and the relative amount of radioactivity was determined by a liquid scintillation counter (Packard, Meriden, CT, USA). Assay sensitivity was 10 pg of CORT per assay tube (0.5 µg/100 mL). The interassay coefficient of variability was calculated, and it was 7.1% (*n* = 4). The plasma LH level was measured by RIA as described [44]. In brief, plasma samples were incubated with ^125^I rat LH and immunoglobulin-G (IgG) antiserum. Rat LH reference standards (NIDDK-rLF-RP-3) were used. The lower limit of detection for this assay is 0.12 ng/mL, and LH values are expressed in relation to the RP-3 standards. The intra-assay and interassay coefficients of variation were 5% and 10%, respectively [44]. Plasma and testicular fluid testosterone concentrations were measured with a tritium-based (^3^H-testosterone) RIA as previously described [45]. In brief, plasma samples (50 µL) or intratesticular fluid samples (5 µL) were incubated overnight with a mixture of rabbit testosterone antiserum and ^3^H-T at 4 °C. No extraction from samples with organic agents was performed. T standards (10–2000 pg/100 µL) were assayed in parallel. Bound steroid was separated from free steroid by mixing with dextran-coated activated charcoal followed by centrifugation. The bound supernatant was put into a bottle with scintillation cocktail, and the relative amount of radioactivity was determined as above. The interassay coefficient of variability was calculated, and it was 7.5% (*n* = 4).

### 4.6. Real-Time PCR (qPCR)

Total RNAs were extracted from the pituitary and testes using TRIzol (Invitrogen, Carlsbad, CA, USA) according to the manufacturer’s instruction. The 12 genes in the testes analyzed and the primers were as described previously [46] (Table S1). Four genes in the pituitary were analyzed, and the primers were listed: (1) *Lhb*, forward primer is 5'-CTGCTGCTGAGCCCAAGTGT-3', and the reverse primer is 5'-TGCTGGTGGTGAAGGTGATG-3'; (2) *Gnrhr*, forward primer is 5'-CTTGAAGCCCGTCCTTGG-3', and the reverse primer is 5'-GCGATCCAGGCTAATCAC-3'; (3) *Esr1*, forward primer is 5'-GCTCCAATTCTGACAATCG-3', and the reverse primer is 5'-TTTCGTATCCCGCCTTTCA-3'; and (4) *Nr3c1*, forward primer is 5'-GAAATGGGCAAAGGCGATAC-3', and the reverse primer is 5'-GCAAATGCCATGAGAAACAT-3'. The relative mRNA levels of targeted genes were normalized to *Rps16* (internal control gene). The RNA was reverse transcribed using random hexamers and MMLV reverse transcriptase (Promega, Madison, CA, USA). QPCR was carried out in a 25 µL volume with SYBR Green. Reactions were carried out, and fluorescence was detected on a Bio-Rad qPCR system (Bio-Rad Laboratories, Inc., Hercules, CA, USA). Each primer was present at 100 nM. The thermal cycle parameters were 94 °C for 15 s (denaturing), 65 °C for 30 s (annealing) and 72 °C for 15 s (extension) for 30 cycles. The *C*t values are provided in Table S1.

### 4.7. Analysis of Steroidogenic Enzyme Activities

HSD3B1, CYP17A1 and HSD17B3 activity were measured. HSD3B1, CYP17A1 and HSD17B3 are only expressed in Leydig cells and not in other testicular cells; Therefore, we used testis homogenates to measure the enzyme activities [47]. A microarray analysis for rat testis samples revealed that rat testis contained HSD3B1, HSD17B3 and HSD17B4, but did not contain HSD3B5 [48]. Therefore, the HSD3B activity in the testis represents the HSD3B1 activities. HSD17B4 is also expressed in the rat testis [49]; However, it behaves as an oxidase, mainly catalyzing estradiol into estrone using NAD^+^ as the cofactor [50]. In the present study, we measured HSD17B reductase activity using NADPH as a cofactor. Therefore, the testis HSD17B reductase activity represents HSD17B3 activity. The activities of HSD3B1, CYP17A1 and HSD17B3 were assayed in testis homogenates, as described previously [51,52]. Three randomly selected samples per group at each time point and one sample per time point in control group were assayed. In brief, rat testis was homogenized, and the homogenate was collected after centrifugation at 750× *g* for 30 min. The concentration of protein (homogenate) was determined using a kit (No. 500-0006, Bio-Rad) with BSA as a standard. The homogenate was incubated for 60 min in the presence of substrates, 2 µM pregnenolone, progesterone or androstenedione, for HSD3B1, CYP17A1 or HSD17B3, and in the presence of 0.2 mM cofactors (NAD^+^ for HSD3B1; NADPH for CYP17A1 and HSD17B3, respectively) and 40 µg homogenate. The reactions were terminated by ice-chilled 1N HCl. The medium levels of progesterone, androstenedione and testosterone were measured by RIA. For the RIAs for progesterone and androstenedione, the commercial kits purchased from IBL were used (IBL, Toronto, ON, Canada), following the manufacturer’s instruction. RIA for testosterone in the medium was performed as described as above. The endogenous steroid in the testis homogenates was also measured to present the background steroid level, which was subtracted to calculate the respective enzyme activities. The formation rate of product was calculated for each enzyme. The *K*m (app) for HSD3B was 0.65 µM, and the Vmax was 32.7 nmol progesterone synthesized/h/mg protein. The *K*m (app) for CYP17A1 was 0.30 µM, and the Vmax was 28.9 nmol androstenedione synthesized /h/mg protein. The *K*m (app) for HSD17B3 was 2.61 µM, and the *V*max was 0.15 nmol testosterone synthesized /h/mg protein.

### 4.8. Western Blot Analysis of StAR

Western blot analysis was conducted. In brief, the homogenized testis samples (10 µg protein, being prepared by the same way described in the section of enzyme assay) of testes were boiled in equal volumes of sample loading buffer, a Tris-Cl buffer (pH 6.8) containing 20% glycerol, 5% SDS, 3.1% dithiothreitol and 0.001% bromophenol blue for 5 min to denature proteins. The samples were then electrophoresed on 10% polyacrylamide gels containing SDS in an electrophoresis system (Bio-Rad) at 80 V. Proteins were electrophoretically transferred onto nitrocellulose membranes, and after 30 min of exposure to 10% non-fat milk to block nonspecific binding, the membranes were incubated with a 1:5000 dilution of a rabbit polyclonal anti-StAR antibody (kindly donated by Stocco, Texas Tech, Lubbock, TX, USA) at 4 °C overnight. The membranes were then washed and incubated with a 1:2500 dilution of goat anti-rabbit antiserum that was conjugated to horseradish peroxidase. The washing step was repeated, and immunoreactive bands were visualized by chemiluminescence using a kit (ECL, Amersham, Arlington Heights, IL, USA). Western blotting analysis of β-Actin (ACTB) using an ACTB antibody (Cell Signaling Technology, Danvers, MA, USA; dilution 1:1,000) was also performed, and the StAR level was normalized to ACTB. The gel was placed in a Kodak image station 4000R (Carestream Health, Inc., Rochester, NY, USA), and photos were taken. The analysis of western blot images was performed using the Kodak image station 4000R. The pixel densities in each band were normalized to the amount of ACTB in each lane. The relative StAR levels to ACTB were calculated.

### 4.9. Statistics

The enzyme activities were transformed to log10. The data of serum hormone, mRNA levels and log10 transformed enzyme activities between control and stress groups during the course of time were analyzed by 2-way ANOVA with time and stress as factors that were corrected with multiple control groups. Sidak’s multiple comparisons test was used to compare each cell mean with the other cell means in between the control and stress group at the same time point. For the analysis of data from *in vitro* testosterone production by Leydig cells, the non-parametric Kolmogorov–Smirnov test was conducted. All data are expressed as the mean ± SEM. Differences were regarded as significant at *p* < 0.05, *p* < 0.01 and *p* < 0.001.

## 5. Conclusions

In summary, the acute immobilization led to the elevated CORT levels as early as 0.5 h followed by the reduced testosterone levels 3 h under immobilization stress. High CORT level may result in binding to NR3C1, which targets cholesterol transport genes (*Scarb1* and *Star*) and steroidogenic enzyme (*Cyp17a1*) genes, to suppress their expressions, which are more sensitive to suppression by acute stress.

## Supplementary Materials

Supplementary materials can be accessed at: http://www.mdpi.com/1422-0067/15/11/21028/s1.
